# Human OVGP1 enhances tyrosine phosphorylation of proteins in the fibrous sheath involving AKAP3 and increases sperm-zona binding

**DOI:** 10.1007/s10815-019-01502-0

**Published:** 2019-06-28

**Authors:** Yuewen Zhao, Frederick W. K. Kan

**Affiliations:** Department of Biomedical and Molecular Sciences, Faculty of Health Sciences, Queen’s University, Kingston, Ontario K7L 3N6 Canada

**Keywords:** In vitro fertilization, Human reproduction, Sperm capacitation, Oviduct, Sperm-egg binding

## Abstract

**Purpose:**

To investigate if the recombinant human oviduct-specific glycoprotein (rHuOVGP1)–enhanced tyrosine-phosphorylated (pY) proteins are components of specific structure(s) of the sperm tail and if rHuOVGP1 binds to the oocyte and enhances sperm-egg binding.

**Methods:**

Immunofluorescent staining and confocal microscopy were performed to examine the localization of pY proteins, outer dense fiber (ODF), and A-Kinase Associated Protein 3 (AKAP3) in human sperm during capacitation. Western blot and immunoprecipitation were employed to analyze protein levels of pY proteins and AKAP3. Immunofluorescent staining was performed to examine the binding of rHuOVGP1 to human oocytes. The effect of rHuOVGP1 on enhancing sperm-zona binding was examined using hemizona assay.

**Results:**

pY proteins were detected mainly in the fibrous sheath (FS) surrounding the ODF with a relatively weak immunoreaction in the neck and mid-piece. Western blot analysis revealed co-migration of the pY 105 kDa protein with AKAP3, which was further confirmed by immunoprecipitation correlating immunofluorescent results of co-localization of pY proteins with AKAP3 in the sperm tail. rHuOVGP1 binds specifically to the zona pellucida (ZP) of human oocytes. Prior incubation of sperm and/or ZP with rHuOVGP1 increased sperm-egg binding.

**Conclusions:**

The present study revealed that one of the major rHuOVGP1-enhanced pY proteins could be AKAP3 of the FS and that rHuOVGP1 is capable of binding to human ZP and its presence in the medium results in an increase in sperm-zona binding. Supplement of rHuOVGP1 in in vitro fertilization media could be beneficial for enhancement of the fertilizing ability of human sperm.

## Introduction

The mammalian oviduct provides an essential microenvironment in its lumen for maturation and transport of gametes, capacitation of sperm, fertilization, and early embryo development [1]. A high-molecular-weight glycoprotein secreted by secretory cells of the oviduct collectively known as oviductin or oviduct-specific glycoprotein (OVGP1) has been identified and characterized in many mammalian species, including the mouse [2, 3], hamster [4], rabbit [5, 6], dog [7], cat [8], sheep [9–11], pig [12], cow [13, 14], rhesus monkey [15], baboon [16, 17], and the human [18]. The amino acid sequence of the N-terminal region of OVGP1 is highly conserved among mammalian species; however, the C-terminal region shows low degree of homology with variable glycosylation sites [19]. Expression of OVGP1 appears to be estrogen-dependent or estrous cycle associated in most species [20–27]. Results from functional studies carried out mainly in vitro indicate several beneficial effects of OVGP1 and the importance of its presence in the luminal milieu of the oviduct during the process of fertilization and early embryo development. For example, bovine OVGP1 was shown to increase the motility and viability of homologous sperm [28] and enhance sperm capacitation and fertilizing capabilities in vitro [29]. The presence of native OVGP1 in the incubation medium was shown to increase sperm-egg binding and penetration rates in the hamster [30], baboon, and human [31] and decrease polyspermy in the pig [32]. Ovine and porcine OVGP1were found to increase cleavage rates of embryos and the number of embryos reaching the blastocyst stage [32, 33]. Previous studies from our laboratory demonstrated that native hamster OVGP1 prepared from the estrus stage can regulate the expression of tyrosine-phosphorylated proteins in hamster sperm during in vitro capacitation [34]. Recombinant hamster OVGP1 has also been found to enhance tyrosine phosphorylation of a subset of sperm proteins during sperm capacitation and increase the number of sperm bound to oocytes [35]. Recombinant human OVGP1 (rHuOVGP1), recently produced in our laboratory, is capable of binding to human sperm, enhancing sperm capacitation through the increase in the level of tyrosine phosphorylation of sperm proteins, and potentiating acrosome reaction [36]. Two of the major tyrosine-phosphorylated proteins that are enhanced by the presence of rHuOVGP1 migrate at 105 kDa (p105) and 81 kDa (p81), respectively [36]. However, the localization and the identity of these tyrosine-phosphorylated proteins during capacitation remained to be elucidated.

The aim of the present study was twofold: firstly, to investigate if the rHuOVGP1-enhanced tyrosine-phosphorylated proteins detected in our previous study are components of specific structure(s) of the sperm tail, and secondly, to investigate if rHuOVGP1 can bind to the zona pellucida (ZP) and enhance sperm-zona binding. The present findings indicate that the major rHuOVGP1-enhanced tyrosine-phosphorylated proteins are associated with A-Kinase Associated Proteins (AKAPs) of the fibrous sheath and that rHuOVGP1 is capable of binding to human ZP in addition to sperm as previously reported. The presence of rHuOVGP1 in the culture medium was also found to increase the number of sperm bound to the oocytes as compared to similar conditions but in its absence.

## Materials and methods

### Materials

The following materials were purchased from the sources indicated: goat anti-human oviductin antibody (P-20), goat anti-AKAP3 antibody (C-20), horseradish peroxidase (HRP)-conjugated donkey anti-goat IgG, goat anti-mouse IgG-HRP, fluorescein isothiocyanate (FITC)-linked goat anti-mouse IgG, donkey anti-goat IgG-CFL555 (Santa Cruz Biotechnology Inc., Santa Cruz, CA, USA), mouse anti-phosphotyrosine antibody (clone 4G10®) (EMD Millipore, Bedford, MA, USA), mouse anti-α-tubulin antibody (clone B-5-1-2), rabbit anti-ODF antibody (Sigma-Aldrich, St. Louis, MO, USA), goat anti-rabbit IgG-Alexa568 antibody (Invitrogen, Burlington, ON, Canada), Western Lighting-Enhanced Chemiluminescence Substrate, and Micromass MassPREP Robotic Protein Handling System (PerkinElmer, Waltham, MA, USA). All other chemicals were obtained from Sigma-Aldrich (St. Louis, MO, USA).

### Production of rHuOVGP1

The production of rHuOVGP1 from stably transfected HEK293 cells in our laboratory using recombinant DNA technology has been described elsewhere [36]. We used a two-step purification system to yield rHuOVGP1 with a purity of ˃ 95%. The resulting protein showed a single band of 120–150 kDa size range on SDS-PAGE corresponding to the molecular mass of the native human OVGP1, and subsequent mass spectrometric analysis of the purified rHuOVGP1 confirmed its identity as human OVGP1 [36].

To determine the optimal concentration of purified rHuOVGP1 for use in in vitro functional studies, a dose-dependent experiment was carried out where human sperm samples were incubated for 4 h in BWW medium supplemented with different concentrations of rHuOVGP1 (i.e., 0, 10, 25, 50, and 75 μg/mL). Western blot analysis was carried out to detect the level of protein tyrosine phosphorylation expression using an anti-phosphotyrosine antibody [36]. Results showed that the strongest labeling intensity of two proteins migrated at 105 kDa and 81 kDa was detected when rHuOVGP1 was used at a concentration of 50 μg/mL [36]. Based on the results obtained from the dose-dependent experiment, rHuOVGP1 at a final concentration of 50 μg/mL was used in all the in vitro functional assays in the present study.

### Semen preparation and sperm capacitation

Studies with human semen were approved by the Ethics Committee for Research on Human Subjects from the Queen’s University Health Sciences and Affiliated Teaching Hospitals Research Ethics Board. Human semen samples were obtained from healthy donors by masturbation after 3 days of sexual abstinence. The sperm samples were prepared and capacitated as previously described [36]. Briefly, after the samples were liquefied in a 37 °C water bath, semen viscosity and volume, sperm motility, and sperm concentration were assessed according to the WHO laboratory manual for the examination and processing of human semen [37]. Normozoospermic samples were selected for experiments in the current study. Liquefied semen samples were separated by centrifugation using 20–40–65–95% Percoll gradients in HEPES buffered saline (HBS; 25 mM HEPES, 130 mM NaCl, 4 mM KCL, 0.4 mM MgCl_2_, 14 mM fructose, pH 7.6). Sperm from the 65–95% interface and in the 95% Percoll fraction representing the highly motile population were pooled and washed with HEPES. Sperm concentration was determined by use of a hemocytometer. Subsequently, sperm were incubated at a concentration of 2 × 10^7^ cells/mL in the presence or absence of 50 μg/mL of recombinant human OVGP1 (rHuOVGP1) for up to 4 h at 37 °C in 5% CO_2_ with 100% humidity in modified Biggers-Whitten-Wittingham medium (BWW; 10 mM HEPES, 94.6 mM NaCl, 4.8 mM KCl. 1.7 mM CaCl_2_, 1.2 mM KH_2_PO_4_, 1.2 mM MgSO_4_, 25.1 mM NaHCO_3_, 5.6 mM d-glucose, 21.6 mM Na lactate, 0.25 mM Na pyruvate, 0.1 mg/mL phenol red, pH 7.4) supplemented with 0.3% fatty acid-free BSA.

### Oocyte collection, storage, and recovery

Studies with human oocytes were approved by the Queen’s University Health Sciences and Affiliated Teaching Hospitals Research Ethics Board (HSREB-Ref. DBMS-030-14). Human oocytes that were immature (GV and MI stage) or not qualified for intracytoplasmic sperm injection procedure were provided by Dr. Tamer Said and obtained with written informed consent from patients at the ReproMed fertility clinic (Toronto, ON, Canada). Oocytes were stored and transported in high salt solution (1.5 M MgCl_2_, 0.1% PVP (MW 40,000), 40 mM sodium HEPES, pH 7.4) at 4 °C. One day before carrying out the experiments, the oocytes were recovered by washing successively in 3 dishes of fresh and pre-equilibrated BWW medium and stored overnight at 37 °C with 5% CO_2_ in droplets of BWW medium covered with oil.

### Oocyte microbisection

Individual oocyte was transferred into mineral oil-covered BWW medium in the absence of BSA and washed in successive droplets of the same medium using oocyte-denuding pipettes until the oocyte settled on the bottom of the culture dish. On the platform of a Leica Wild M3Z inverted microscope (Leica Microsystems, Heerbrugg, Switzerland), a glass blade with a sharp angled-tip, held by hand, was lowered vertically into the medium droplet. By pressing down the glass blade steadily and firmly in the median line of the oocyte and moving along the cutting axis, the oocyte was bisected into two equal halves. Subsequently, the hemizona pairs were washed using denuding pipettes to remove the residual cytoplasm. Mineral oil was removed and the hemizona pairs were transferred into BWW medium droplets supplemented with BSA. The hemizona pairs were incubated overnight at 37 °C with 5% CO_2_ in BWW medium droplets covered with mineral oil_._

### Hemizona assay

Hemizona assay was performed following the procedure previously described by Oehninger et al. [38] with modifications. Three different experimental conditions were carried out with 8–9 oocytes used in each condition to examine the effect of rHuOVGP1 on enhancement of sperm-zona binding. (1) Pre-treatment of sperm and hemizonae, respectively, with rHuOVGP1: hemizonae were incubated in BWW medium droplets in the presence of 50 μg/mL rHuOVGP1 for 1 h at 37 °C with 5% CO_2_. Sperm were suspended at a concentration of 2 × 10^6^/mL in BWW medium in the presence of 50 μg/mL rHuOVGP1. (2) Pre-treatment of sperm with rHuOVGP1: sperm were incubated in BWW medium in the presence of 50 μg/mL rHuOVGP1 and incubated at 37 °C with 5% CO_2_ for 1 h. The sperm sample was washed with HBS solution followed by centrifugation. The sperm sample was then diluted in fresh BWW medium at a concentration of 2 × 10^6^/mL. Hemizonae were used without prior treatment with rHuOVGP1. (3) Pre-treatment of hemizonae with rHuOVGP1: hemizonae were incubated in BWW medium droplets in the presence of 50 μg/mL rHuOVGP1 for 1 h at 37 °C with 5% CO_2_. Sperm suspension was prepared at a concentration of 2 × 10^6^/mL in fresh BWW medium without prior treatment with rHuOVGP1. In the experiments above, sperm and hemizonae were incubated in the presence of rHuOVGP1 for 1 h because, in a previous study where we reported the production and purification of recombinant hamster OVGP1 (rHamOVGP1), we successfully used 1 h for incubation in the sperm-oocyte binding assay in the presence of rHamOVGP1 [35]. Furthermore, in our earlier report on the successful production and purification of rHuOVGP1, the binding of rHuOVGP1 to the head, the mid-piece, and the principal piece of human sperm was evident at 1 h of in vitro capacitation in the presence of rHuOVGP1 [36]. Therefore, based on these premises [35, 36], 1-h incubation was used in the hemizona assay. For all experiments, droplets of 25 μL of sperm suspension were prepared on a culture dish and covered with mineral oil. Hemizonae were added into the sperm droplets and incubated for 4 h at 37 °C with 5% CO_2_. Control hemizonae were incubated in BWW medium in the absence of rHuOVGP1, transferred into sperm droplets in BWW medium in the absence of rHuOVGP1, and incubated in the same manner as described above.

### Hemizona index analysis

Following sperm-hemizona incubation, the hemizonae were washed 5 times in PBS and then transferred into 1% paraformaldehyde (PFA)-PBS droplets covered with oil. Photomicrographs were captured with different focal planes that covered all zona-bound sperm. The number of bound sperm from all focal planes was counted and hemizona index (HZI) was calculated using the equation below [38]. If the total number of sperm was < 20 for the hemizona or the hemizona was saturated with sperm, the assay results for that particular experimental hemizona and the control half were not included when reporting results.

$$ \mathrm{HZI}=\frac{\mathrm{Number}\ \mathrm{of}\ \mathrm{sperm}\ \mathrm{bound}\ \mathrm{in}\ \mathrm{the}\ \mathrm{test}\ \mathrm{sample}}{\mathrm{Number}\ \mathrm{of}\ \mathrm{bound}\ \mathrm{sperm}\ \mathrm{in}\ \mathrm{the}\ \mathrm{control}\ \mathrm{sample}}\times 100 $$

### Immunofluorescent imaging of binding of rHuOVGP1 to oocytes

Oocytes were recovered and incubated in pre-equilibrated BWW medium overnight at 37 °C with 5% CO_2_. The oocytes were then transferred into BWW medium droplets covered with mineral oil in the presence or absence of 50 μg/mL rHuOVGP1 and incubated for 4 h at 37 °C with 5% CO_2_. After incubation, oocytes were washed in PBS and then fixed in 1% PFA-PBS for 30 min at room temperature. The oocytes were washed with PBS and transferred into oil-covered droplets of 5% donkey serum-PBS and incubated for 30 min. Subsequently, the oocytes were incubated at 4 °C overnight with goat polyclonal anti-OVGP1 antibody at a concentration of 2 μg/mL in 30 μL of 1% donkey serum-PBS covered with oil. Thereafter, the oocytes were washed with PBS and incubated with donkey anti-goat IgG-FITC antibody at a concentration of 1 μg/mL in 1% donkey serum-PBS for 1 h at room temperature in the dark. The oocytes were then washed with PBS, transferred onto glass slides, and mounted with 1% 1,4-Diazabicyclo-(2,2,2)-octane (DABCO) in PBS with 90% glycerol. Images of oocytes were captured using a Zeiss Axiovert 200M Fluorescence Imaging Microscope (Carl Zeiss Meditec, Oberkochen, Germany).

### Immunofluorescent labeling of sperm

Sperm samples were capacitated in BWW medium in the presence or absence of 50 μg/mL rHuOVGP1, washed with HBS, and then spread on positively charged glass slides. For detecting tyrosine-phosphorylated (pY) proteins, sperm samples on slides were fixed with 1% PFA-PBS for 30 min and washed with PBS, treated with 90% ethanol for 30 min in a humid chamber, and followed by PBS washes. The sperm slides were first incubated with 10% normal goat serum (NGS)-PBS containing 0.05% Tween-20 (NGS-PBST) for 1 h at room temperature followed by incubation with mouse anti-phosphotyrosine antibody (0.5 μg/mL) in 5% NGS-PBST at 4 °C overnight. Subsequently, the slides were washed with PBST and then incubated with goat anti-mouse IgG-FITC antibody (1 μg/mL) in 5% NGS-PBST at room temperature for 1 h. After being washed with PBST and mounted with 1% DABCO in PBS and 90% glycerol, the slides were examined on a Zeiss Axiovert 200M Fluorescence Imaging Microscope. For confocal microscopic examination of the localization of pY proteins and outer dense fiber (ODF), sperm samples were extracted with Triton-DTT buffer (2% TritonX-100, 5 mM DTT, 50 mM Tris-HCl, pH 9.0, and protease inhibitors) for 15 min at room temperature with agitation. Extracted samples were centrifuged and washed with Tris-HCl (pH 9.0). The pellets were resuspended with PBS and spread on glass slides. The cell smear was fixed immediately and washed. The sperm slides were then blocked with 10% NGS-PBST followed by incubation with mouse anti-phosphotyrosine antibody (0.5 μg/mL) and rabbit anti-ODF antibody (2 μg/mL) in 5% NGS-PBST, and subsequently with goat anti-mouse IgG-FITC antibody (1 μg/mL) and goat anti-rabbit IgG-Alexa568 antibody (8 μg/mL) in 5% NGS-PBST for 1 h following the procedure as mentioned earlier. After the successive incubations, the sperm slides were washed with PBS, mounted with DABCO, and examined on a Leica TCS SP2 confocal microscope (TCS-MP, Heidelberg, Germany).

For co-localization of AKAP3 and pY proteins, the sperm sample was first divided into a membrane-intact fraction and fixed with 1% PFA-PBS after capacitation and a membrane-extracted fraction that was first treated with Triton-DTT buffer and then fixed with 1% PFA-PBS. In each case, after preparing sperm smears on glass slides as described above, sperm slides were treated with 90% ethanol, washed with PBS, and blocked with 3% BSA-PBST for 1 h at room temperature followed by incubation overnight at 4 °C with goat anti-AKAP3 antibody (4 μg/mL) and mouse anti-phosphotyrosine antibody (0.5 μg/mL) in 1% BSA-PBST. After being washed with PBST, the sperm slides were incubated with donkey anti-goat IgG-CFL555 (1 μg/mL) in 1% BSA-PBST for 1 h at room temperature followed by several washes with PBST. The sperm slides were then incubated with goat anti-mouse IgG-FITC (1 μg/mL) in 1% BSA-PBST for 1 h at room temperature, washed with PBST, and mounted with DABCO. Fluorescent images were captured using a Leica TCS-SP2 laser scanning multiphoton confocal microscope (Leica Microsystem, Heidelberg, Germany).

### Western blot analysis

Detection of the level of protein tyrosine phosphorylation and AKAP3 protein expression was carried out as previously described [36]. Briefly, capacitated and non-capacitated sperm samples were solubilized in electrophoresis sample buffer (2% SDS, 10% glycerol, 50 mM dithiothreitol, 62.5 mM Tris-HCl, pH 6.8) and boiled at 100 °C for 5 min. Sperm proteins were resolved by SDS-PAGE and transferred onto a PVDF membrane. To compare the migration distance of the pY proteins and AKAP3, the membrane was cut along the mid-line of the lane with sperm sample that was capacitated for 4 h in the presence of rHuOVGP1. The membrane halves were blocked with 5% (*w*/*v*) skim milk in Tris-buffered saline (150 mM NaCl, 50 mM Tris, pH 7.5) containing 0.05% Tween 20 (TBST). Half of the membrane was incubated with mouse monoclonal anti-phosphotyrosine antibody (0.1 μg/mL in TBST) for 1 h at room temperature followed by several washes with TBST. The membrane was then incubated for 1 h with goat anti-mouse IgG-HRP (0.02 μg/mL) in TBST containing 5% skim milk and washed with TBST. The other half of the membrane was incubated overnight at 4 °C with goat anti-AKAP3 antibody (1 μg/mL in TBST containing 5% skim milk). After incubation, the membrane was washed several times with TBST and blotted with donkey anti-goat IgG-HRP (0.1 μg/mL in TBST with 5% skim milk) for 1 h followed by several washes with TBST. The two halves of the membrane were matched and aligned along the cutting edge, and the immunoreactivity was revealed using the Electrogenerated chemiluminescence (ECL) kit according to the manufacturer’s instruction. The same membranes were probed with mouse monoclonal anti-α-tubulin antibody (0.1 μg/mL in TBST) for 30 min at room temperature followed by goat anti-mouse IgG-HRP (0.02 μg/mL). The intensities of immunoreaction were quantified using ImageJ software. Intensities of pY proteins and AKAP3 were normalized to the ones of α-tubulin.

### Immunoprecipitation of AKAP3

Immunoprecipitation of AKAP3 was performed as previously described by Luconi et al, with modifications [39]. Briefly, each sample containing 50 million of sperm cells was subjected to capacitation. Sperm samples were lysed in SDS-lysis buffer (20 mM Tris-HCl, pH 7.4, 1% SDS, 1 mM PMSF, 1 mM Na_3_VO_4_) by vortexing for 1 min and then rotating for 30 min at 4 °C. After 5 min of boiling at 95 °C, samples were kept for 10 min on ice and centrifuged at 16,000×*g* for 5 min at room temperature. Extracted supernatants were diluted 10 times with 20 mM Tris-HCl, pH 7.4 buffer and subjected to AKAP3 immunoprecipitation by first incubating overnight using 1.5 μg of goat anti-AKAP3 antibody followed by 2 h of incubation at 4 °C with 15 μL of protein G-Sepharose beads. The protein beads were washed three times in 20 mM Tris-HCl, pH 7.4 by centrifugation at 1000×*g* at 4 °C for 5 min and then resuspended in sample buffer and subjected to SDS-PAGE followed by Western blot analysis.

### Statistical analysis

Statistical analysis was performed using GraphPad software PRISM. Data are expressed as mean ± SE. *P* values equal to or less than 0.05 with 95% confidence intervals were considered statistically significant.

## Results

### Tyrosine-phosphorylated proteins are predominantly localized in the fibrous sheath (FS) of the sperm tail

**Fig. 1:**
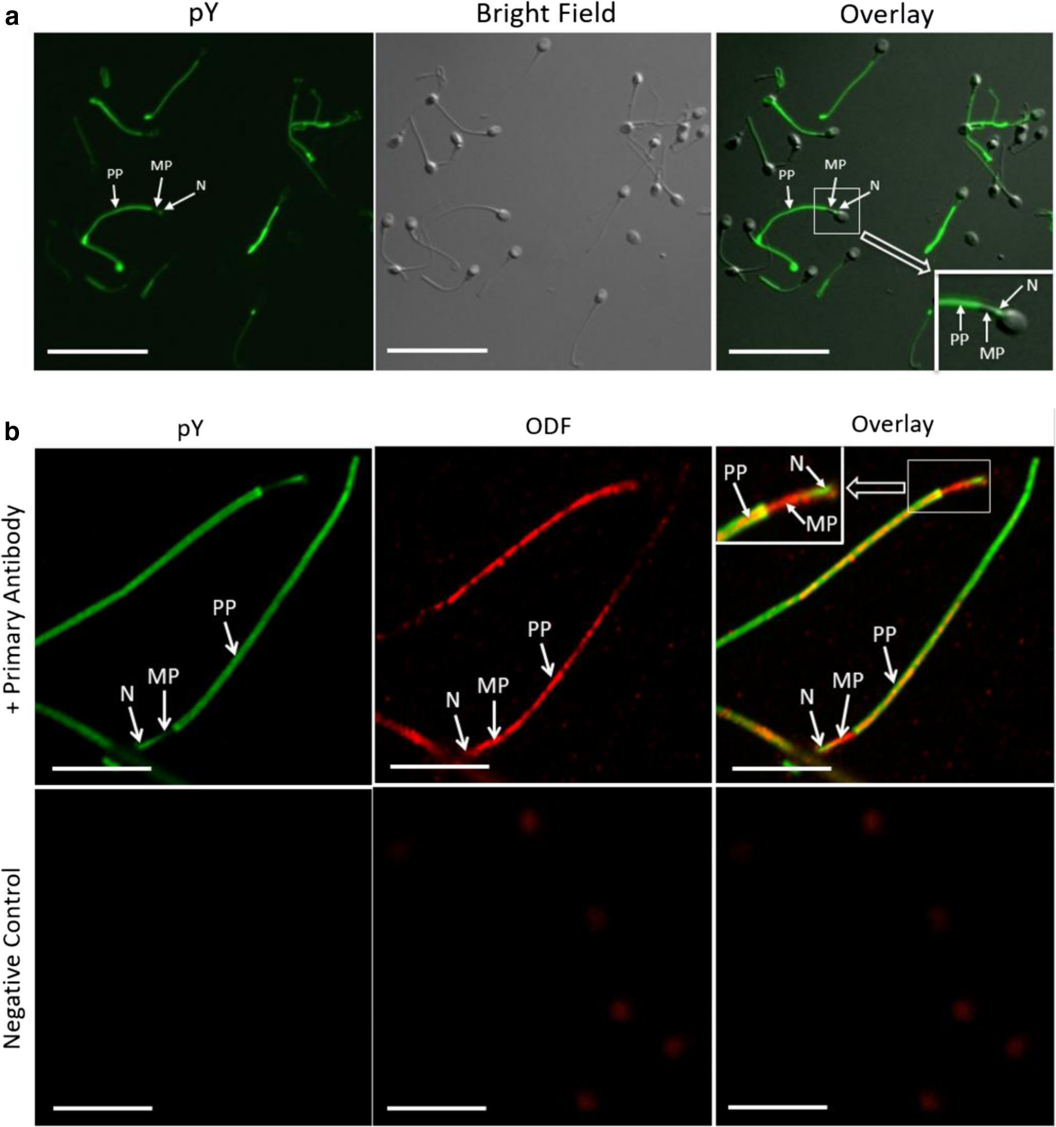
Tyrosine-phosphorylated proteins are predominantly localized to the fibrous sheath of the sperm tail. **a** Microscopic images showing the immunofluorescent labeling of sperm proteins that are tyrosine phosphorylated (pY) following capacitation. Scale bar = 50 μm. **b** Confocal fluorescent images showing the immunofluorescent labeling of tyrosine-phosphorylated sperm proteins (upper left), the ODF protein (upper middle), the overlay image (upper right), and corresponding negative controls (lower panel). Insets: high magnifications of sperm cells within the framed boxes revealing various immunolabeled structures. Scale bar = 10 μm. N, neck; MP, mid-piece; PP, principal piece

One characteristic feature during sperm capacitation is the time-dependent increased level of tyrosine phosphorylation of sperm proteins [40–42]. We have previously shown that supplement of rHuOVGP1 at an optimal concentration of 50 μg/ml in the capacitating medium can significantly increase the level of tyrosine phosphorylation of a subset of proteins in human sperm [36]. In order to further investigate the role of rHuOVGP1 in regulating human sperm function, immunofluorescent experiments were carried out in the present study to examine the specific site of localization of pY proteins following capacitation. At least 200 sperm cells were examined on each slide. Photomicrographs representative of the immuno-detection of pY proteins in human sperm are best illustrated in Fig. 1a where immunofluorescent and overlay images showed that the tyrosine-phosphorylated (pY) proteins were predominantly localized in the principal piece of the sperm tail. A relatively weak immunoreactivity for pY proteins was also detected in the neck region immediately below the sperm head with no labeling in the mid-piece region.

As the immunoreactivity for the pY proteins was found to be mainly associated with the principal piece of the sperm tail, the possible association of pY proteins with the underlying cytoskeletal elements in the tail region was further explored by removing the sperm plasma membrane (PM) and mitochondrial sheath (MS) using Triton-DTT extraction buffer followed by co-localization of the pY proteins and the outer dense fiber (ODF) (Fig. 1b). The pattern of immunolabeling of pY proteins in sperm after extraction of PM and MS (Fig. 1b: pY) was similar to that of the intact sperm in the tail region (Fig. 1a: pY). After extraction with Triton-DTT, the immunoreactivity remained with the entire principal piece of the tail (Fig. 1b: pY). The labeling appeared to be stronger along the outer edges and weaker along the mid-line of the principal piece. The neck region immediately below the sperm head was strongly labeled and the immunoreactivity gradually decreased towards the mid-piece region (Fig. 1b: pY). Immunolabeling of ODF, however, displayed a different pattern than that of pY proteins. In general, immunoreactivity of ODF was detected in all sperm examined with an intact tail. ODF was found throughout the neck region, mid-piece, and principal piece, albeit with a higher intensity in the neck region and the proximal segment of the mid-piece (Fig. 1b: ODF). The intensity of immunoreaction decreased towards the end of principal piece. The overlay image of the immunolabeling of pY proteins and ODF revealed that the mid-piece houses the proximal segment of the ODF but was devoid of pY proteins. The pY proteins and ODF were co-localized along the mid-line of the principal piece where ODF is located. However, the majority of the pY proteins were localized to the periphery of the principal piece where the FS is present. The pY proteins appeared to form a sheath in the principal piece enclosing the ODF (Fig. 1b: Overlay). The immunostaining pattern described above was similar in all sperm cells examined. One characteristic feature of the principal piece is the FS that surrounds the bundle of ODF. Therefore, the majority of the up-regulated pY proteins following capacitation are likely associated with the FS of the sperm tail. The immunofluorescent signals were essentially absent in the negative controls for both pY proteins and ODF (Fig. 1b: lower panel).

### rHuOVGP1 enhances tyrosine phosphorylation of AKAP3 located in the fibrous sheath

**Fig. 2:**
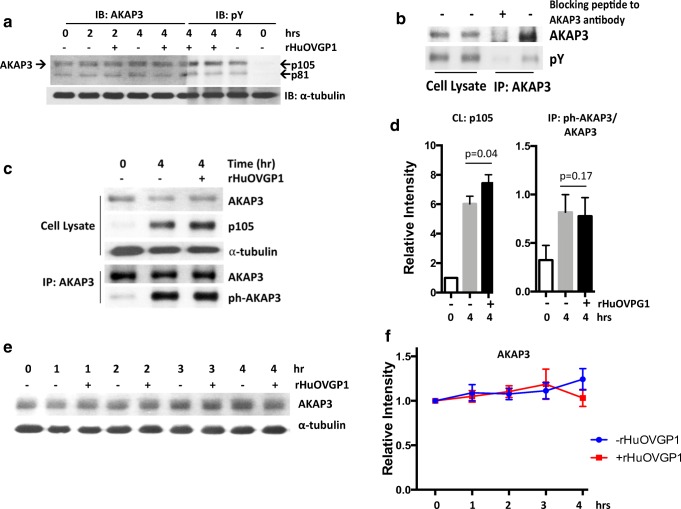
The effect of rHuOVGP1 on tyrosine phosphorylation of AKAP3. **a** Western blot showing co-migration of AKAP3 (left) and tyrosine-phosphorylated proteins (PY: right) from human sperm after incubation in capacitating medium in the presence or absence of 50 μg/mL rHuOVGP1 from 0–4 h. Lower panel shows the immunoblotting of α-tubulin. **b** Experiment of AKAP3 immunoprecipitation where sperm extracts were immunoprecipitated using anti-AKAP3 antibody alone or in the presence of a blocking peptide specific for the anti-AKAP3 antibody. Western blot analysis using anti-AKAP3 antibody (AKAP3) or stripping and re-probing with anti-phosphotyrosine antibody (p105) is shown. **c** Comparison of the levels of AKAP3 and tyrosine-phosphorylated p105 in the presence or absence of 50 μg/mL rHuOVGP1 following 4 h of capacitation in the sperm cell lysate as well as the levels of immunoprecipitated AKAP3 and the tyrosine-phosphorylated AKAP3 (ph-AKAP3) from the respective sperm cell lysates. **d** Statistical analysis of the intensities of protein bands from Western blots showing the levels of AKAP3 in cell lysates, p105 in cell lysates, and ph-AKAP3/AKAP3 in the eluates of immunoprecipitation (data represent means ± SEM obtained from four separate experiments). **e** Western blot showing the levels of AKAP3 following capacitation in the presence or absence of 50 μg/mL rHuOVGP1. **f** Statistical analysis of the intensities of protein bands from Western blots showing the protein level of AKAP3 during 4 h of capacitation in the presence (blue) or absence (red) of rHuOVGP1 (data represent means ± SEM obtained from seven separate experiments)

We previously reported that one of the most abundantly tyrosine-phosphorylated human sperm proteins migrates at 105 kDa (p105), the phosphorylation level of which was further enhanced by the presence of optimally 50 μg/mL rHuOVGP1 in the capacitating medium [36]. Based on our previous results and the initial findings of association of pY proteins with the FS as well as current information available in the literature concerning proteins in the FS [43–46], we postulated that the p105 could be A-Kinase Anchoring Protein 3 (AKAP3). Western blot analysis was carried out to examine whether, in human sperm, AKAP3 co-migrates with the tyrosine-phosphorylated p105. As shown in Fig. 2a, two protein bands were detected in the sperm protein lysate using goat anti-AKAP3 antibody. The protein band corresponding to AKAP3 co-migrated with p105 at the same distance, indicating that the tyrosine-phosphorylated p105 during capacitation that we previously reported is likely the AKAP3. A second protein band with a weaker immunoreactivity co-migrated with tyrosine-phosphorylated p81 (Fig. 2a). Based on the sequence similarities at the C-terminal regions of AKAP3 and AKAP4 and the fact that AKAP4 is known to be tyrosine-phosphorylated during capacitation [47], the tyrosine-phosphorylated p81 that we previously reported is likely AKAP4. Immunoprecipitation experiments were carried out to determine the identity of p105 using goat anti-AKAP3 antibody (Fig. 2b). The anti-AKAP3 antibody was able to sufficiently pull down AKAP3 from the sperm lysate. Stripping and re-probing the same membrane with anti-phosphotyrosine antibody showed that the immunoprecipitated AKAP3 was also tyrosine phosphorylated that migrated at the same level as compared to p105 in the whole cell lysate. The blocking peptide for the anti-AKAP3 antibody successfully blocked the immunoprecipitation of AKAP3 as well as the tyrosine phosphorylation signals from AKAP3 (Fig. 2b). Having shown that the 105 kDa protein (p105) corresponds to the tyrosine-phosphorylated AKAP3, further experiment was carried out to determine the effect of rHuOVGP1 on the tyrosine phosphorylation level of AKAP3 following capacitation. Similar to results that we recently reported [36], in the present study, the tyrosine phosphorylation level of p105 in the cell lysate was found to be increased in sperm capacitated in the presence of rHuOVGP1 as compared to sperm capacitated in the absence of rHuOVGP1 (*P* = 0.04, Fig. 2c, d). For the uncapacitated sperm, the tyrosine phosphorylation level of AKAP3 (ph-AKAP3) was found to be very low (Fig. 2c, d). Following 4 h of capacitation, the level of ph-AKAP3 was largely increased. However, in the presence of rHuOVGP1, the level of ph-AKAP3 was found to be about the same as compared to sperm capacitated in the absence of rHuOVGP1 (*P* = 0.17, Fig. 2c, d).

Although a trend of increase of AKAP3 protein levels with a peak at 4 h in the absence of rHuOVGP1 and a peak at 3 h in the presence of rHuOVGP1 was noted, the protein levels of AKAP3 are not statistically different between the hours of capacitation (Fig. 2e, f). A marginal decrease of AKAP3 level at 4 h of capacitation in the presence of rHuOVGP1 was observed as compared to capacitation in the absence of rHuOVGP1 (*P* = 0.059, Fig. 2f). Surely, a larger sample size has to be used in the future to determine unequivocally whether these observed trends are meaningful.

**Fig. 3:**
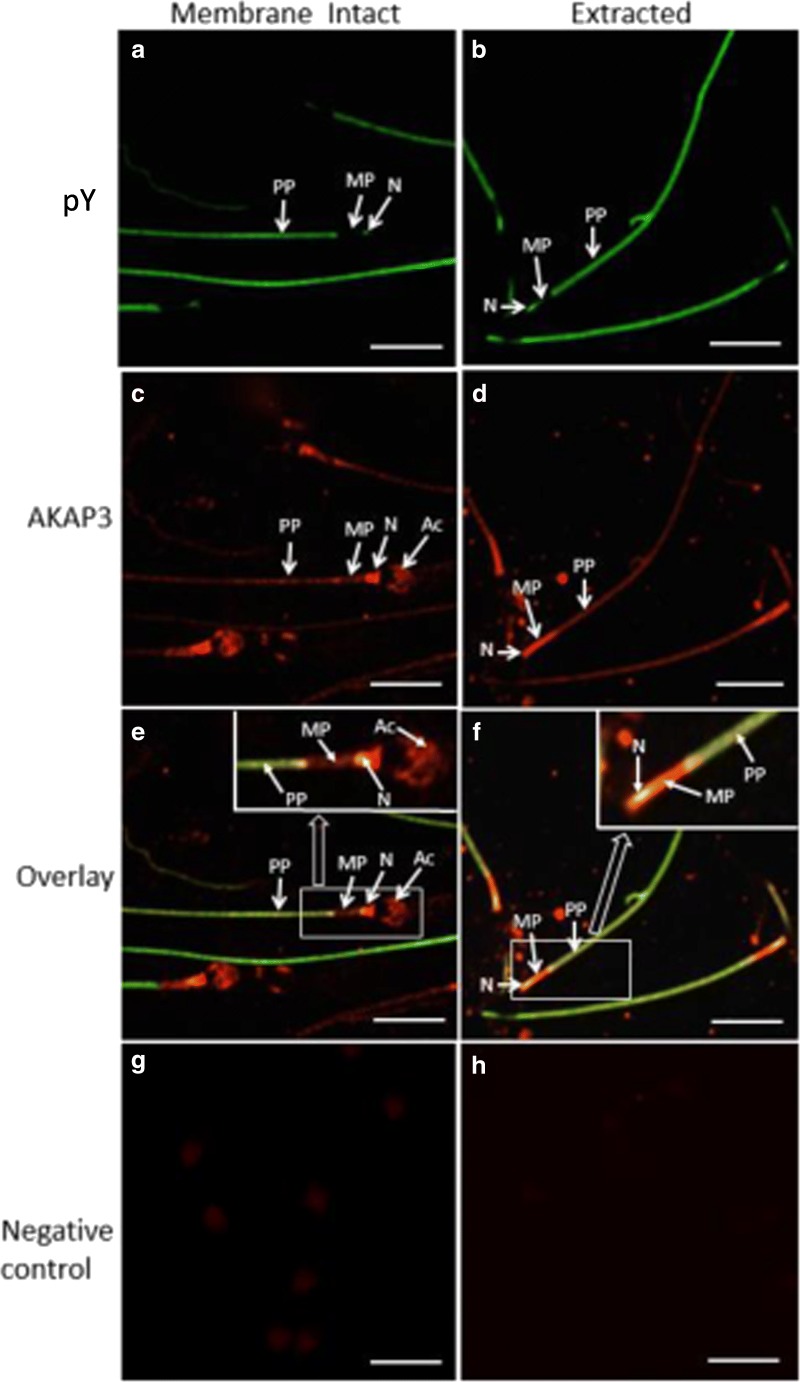
AKAP3 is co-localized with pY proteins in the neck region and in the principal piece of the sperm tail. Confocal microscopic images of immunofluorescent labeling of pY proteins (a and b) and AKAP3 (c and d) on capacitated sperm with intact membrane (a, c, and e) and Triton-DTT extracted (b, d, and f) sperm. Bottom panel (g and h) shows the negative control of AKAP3 labeling. Insets: high magnifications of sperm cells within the framed boxes revealing various immunolabeled structures. Scale bar = 10 μm. N, neck region; MP, mid-piece, PP, principal piece; Ac, acrosome

To further examine whether AKAP proteins co-localized with pY proteins in human sperm, double immunostaining experiments were performed using anti-AKAP3 and anti-phosphotyrosine antibodies. Both membrane-intact and Triton-DTT extracted sperm were examined for AKAP3 and pY co-localization. At least 200 sperm cells were examined in each condition. Whereas all sperm examined having an intact tail structure displayed immunoreactivity of AKAP3, the intensity of immunostaining for tyrosine phosphorylation varied among sperm cells. In membrane-intact sperm, immunoreaction for pY was found to be specifically associated with the neck region and the entire principal piece of the tail (Fig. 3a). After extraction with Triton-DTT, immunoreaction over the neck region became more intense and extended into the mid-piece while the staining pattern remained the same in the principal piece before and after extraction (Fig. 3b). In membrane-intact sperm, immunoreactivity of AKAP3 was found in the acrosomal region, the neck region, the mid-piece and the principal piece (Fig. 3c). After extraction with Triton-DTT, immunoreactivity was absent in the sperm head and became weaker in the neck region (Fig. 3d). Immunostaining for AKAP3 persisted in the tail with an intense and uniformly distributed immunoreaction over the mid-piece after extraction with Triton-DTT (Fig. 3d). The overlay image demonstrated a high degree of co-localization of AKAP3 and pY proteins in the neck region and in the principal piece of sperm with or without Triton-DTT extraction. However, the co-localization appeared to be more pronounced in the neck region after treatment with Triton-DTT (Fig. 3: compare f with e). The previously observed immunoreactivity in the membrane-intact sperm and in the Triton-DTT extracted sperm was abolished when a blocking peptide specific for anti-AKAP3 antibody was added to the antibody prior to immunostaining (Fig. 3g, h). Based on these findings, the detergent-soluble AKAP3 in the mid-piece and in the acrosome region does not appear to be co-localized with tyrosine-phosphorylated proteins, whereas the opposite is true of detergent-insoluble AKAP3 associated with the neck region and the principal piece.

### rHuOVGP1 binds to the ZP of oocytes

**Fig. 4:**
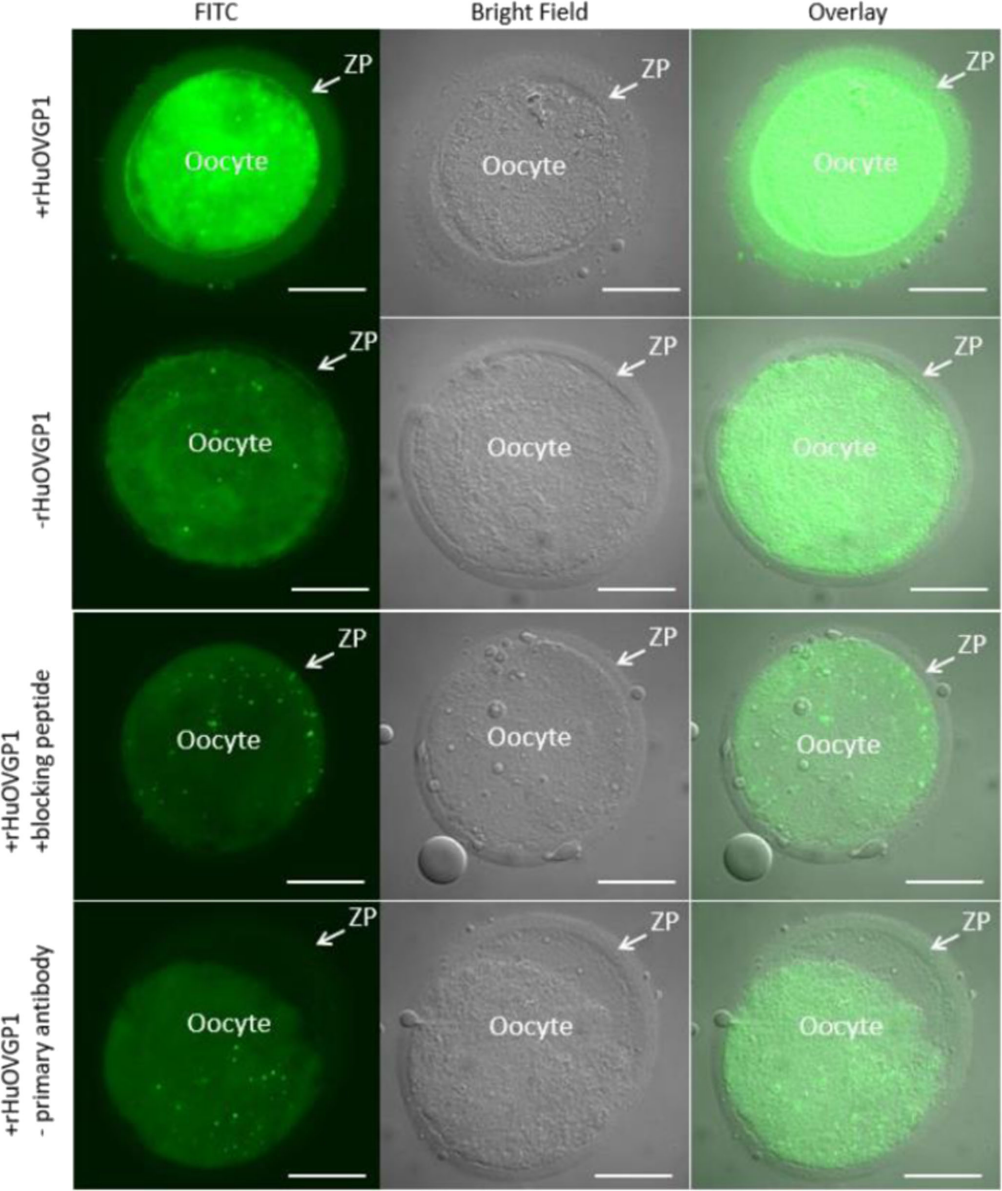
rHuOVGP1 binds to the ZP of human oocytes. Microscopic images showing the immunofluorescent labeling of rHuOVGP1 in human oocytes following incubation of oocytes in the presence (+) or absence (−) of rHuOVGP1, with the blocking peptide specific for the OVGP1 antibody, or in the absence (−) of the primary antibody during immunolabeling, respectively. Scale bar = 50 μm

Following our previous study reporting that rHuOVGP1 binds to the cell surface and underlying cytoskeletal elements of human sperm [36], in the present study, we went further to investigate the effect of rHuOVGP1 on early events of fertilization. We first examined the ability of rHuOVGP1 to bind to the zona pellucida (ZP) of human oocytes. Human oocytes recovered from high-salt storage buffer and incubated in BWW in the presence of 50 μg/mL of rHuOVGP1 followed by labeling with anti-human OVGP1 antibody showed immunoreaction uniformly distributed throughout the ZP surrounding the oocyte (Fig. 4). The oocyte cytoplasm was also immunoreactive to the antibody. However, the cytoplasmic immunoreaction was later found to be non-specific since oocytes incubated in BWW in the absence of rHuOVGP1 also showed similar non-specific immunoreactivity in the oocyte cytoplasm whereas the ZP displayed no immunoreactivity (Fig. 4). The ZP was also devoid of immunostaining in negative controls where human oocytes were incubated with rHuOVGP1 in the presence of a specific blocking peptide or where oocytes were incubated with rHuOVGP1 but with the omission of the primary antibody. Therefore, our results demonstrated the capability of rHuOVGP1 in binding to the ZP of human oocytes in addition to human sperm as we previously reported [36]. Immunostaining of the oocyte cytoplasm is considered non-specific due to a false-positive immunoreaction from the secondary antibody.

### rHuOVGP1 increases the number of sperm bound to ZP

**Fig. 5:**
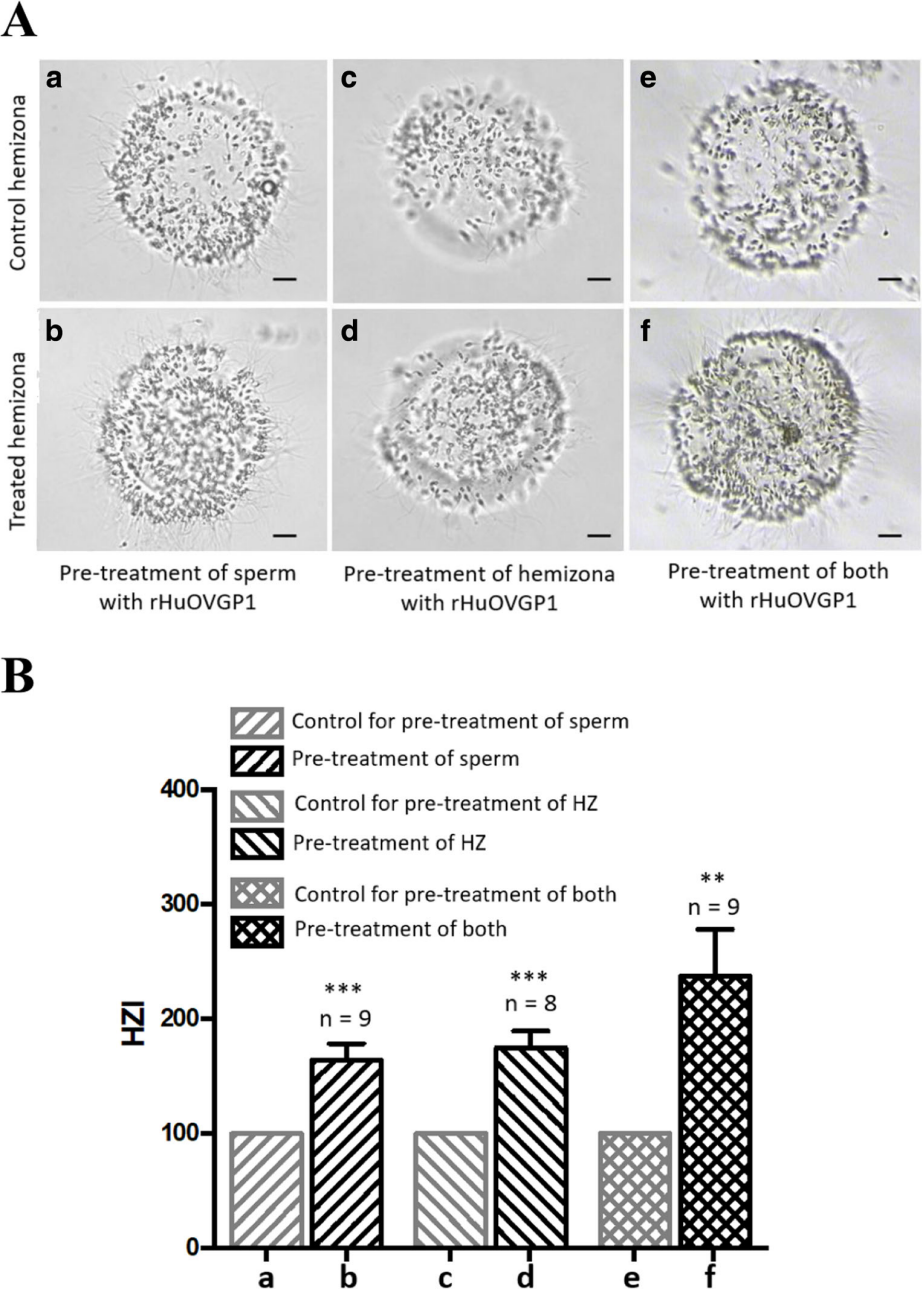
Effects of rHuOVGP1 on sperm-ZP binding. **A** Representative microscopic images showing the hemizona binding assays from the three experimental conditions: (b) pre-treatment of sperm with rHuOVGP1, (d) pre-treatment of hemizonae with rHuOVGP1, and (f) pre-treatment of both sperm and hemizonae with rHuOVGP1, as compared to their corresponding control assays (a, c, and e) in the absence of rHuOVGP1. **B** Histogram showing statistical analysis of hemizona index of the aforementioned assays. Data are shown as the mean ± SEM; *n* indicates the number of hemizona pairs for each experimental condition; **P < 0.005; ***P < 0.001

Carried forward from our previous study showing that rHuOVGP1 can potentiate acrosome reaction [36], the role of rHuOVGP1 in sperm-egg binding was investigated in the present study by performing three different experimental conditions of hemizona binding assay (HZA). Treatment of both the sperm and hemizonae with 50 μg/mL of rHuOVGP1 prior to HZA yielded an increase of the hemizona index (HZI) by 2.37-fold (*P* < 0.005) as compared to the untreated conditions (Fig. 5). The HZI was increased by 1.64-fold (*P* < 0.001) when sperm were treated with 50 μg/mL of rHuOVGP1 prior to incubation with the untreated hemizonae as compared to that of the untreated sperm incubated with untreated hemizonae (Fig. 5). Similarly, prior treatment of the hemizonae with rHuOVGP1 before incubation with untreated sperm yielded an increase in the HZI by 1.75-fold (*P* < 0.001) as compared to the untreated hemizonae (Fig. 5). As a result, pre-treatment of sperm and/or ZP can enhance the number of sperm bound to ZP albeit at different levels.

### Discussion

During capacitation in human sperm, protein tyrosine phosphorylation in the principal piece but not the acrosome, equatorial segment, or the neck region is correlated with fertilization rates [48]. However, the subcellular localization and identity of these tyrosine-phosphorylated (pY) proteins in human sperm remained to be elucidated. Recently, we have shown that during in vitro capacitation, the presence of rHuOVGP1 in the capacitating medium can further enhance the level of tyrosine phosphorylation of two sperm proteins migrated at 105 kDa and 81 kDa, respectively, on SDS-PAGE [36]. In the present study, we further demonstrated that the tyrosine-phosphorylated proteins are predominantly localized in the fibrous sheath (FS) of the principal piece of the sperm tail in both membrane-intact and Triton-DTT-treated sperm. A relatively weak immunofluorescent signal was also found in the neck region of the membrane-intact sperm. Interestingly, after extraction with Triton-DTT, the intensity of immunostaining of pY proteins was found to increase in the neck region, and the immunoreactivity extended towards the mid-piece. In human sperm, a tightly packed and well-organized mitochondrial sheath envelops the cytoskeletal tubules of the mid-piece. Triton-DTT treatment has been used to remove the plasma membrane and the underlying mitochondria in the mid-piece of mouse sperm [49]. Removal of the plasma membrane and mitochondrial sheath allows the anti-phosphotyrosine antibody to gain access to the underlying pY proteins in the mid-piece as shown in the present study. Our results showed that immunostaining for the outer dense fiber (ODF) is present throughout the neck region, mid-piece, and principal piece of the sperm tail. The finding of immunoreactivity of ODF decreasing towards the end piece is in agreement with earlier findings that the majority of the ODF extends from the mid-piece downward to reach up to 60% of the entire length of the principal piece [50]. Overlay immunofluorescent images of pY proteins and ODF showed co-localization of pY proteins with ODF in the mid-line of principal piece but not in the neck region or in the mid-piece. In the principal piece, the immunostaining of pY proteins appeared to be weaker in the mid-line but intensely stained along the outer edges, forming a sheath surrounding the ODF. This suggests that the pY proteins are predominantly localized to the FS of the sperm tail. In the rat and hamster, the outer dense fiber protein has been reported to be tyrosine-phosphorylated during sperm capacitation [51, 52]. The present results showing co-localization of pY proteins and ODF in the principal piece of human sperm during capacitation corroborates with observations made in the rat and hamster [51, 52].

Previous proteomic studies have revealed that the proteins undergoing tyrosine phosphorylation during sperm capacitation include ion channels, metabolic enzymes, and structural proteins [51, 53, 54]. Two prominent tyrosine-phosphorylated proteins are A-kinase anchoring protein (AKAP) 3 and 4 [51]. Phosphorylated AKAPs sequester a cyclic adenosine monophosphate (cAMP)-dependent protein kinase A (PKA), a key enzyme which induces sperm capacitation, to subcellular compartments by binding to the regulatory subunit RII of PKA [55–57]. AKAP3 and 4 are known to be the main components in the FS of the sperm tail [43–46]. In the present study, AKAP3 was found to co-migrate with the tyrosine-phosphorylated p105 (Fig. 2a). The AKAP3 antibody, produced against the C-terminus of human AKAP3 protein, also detected a lower band that co-migrated with p81. The C-terminus of human AKAP3 and AKAP4 shares a homology domain with high protein sequence identity (NCBI Reference Sequence: NP_001265238.2 and NP_003877.2). It is likely that the same antibody can also detect AKAP4 protein. In the present study, immunoprecipitation experiment using anti-AKAP3 antibody showed that the tyrosine-phosphorylated sperm protein p105 corresponds to AKAP3, which is in agreement with the findings reported by Luconi et al. [39] that AKAP3 is the major tyrosine-phosphorylated sperm protein following capacitation. However, contrary to a significant increase in the level of tyrosine-phosphorylated p105 at 4 h of capacitation in the presence of rHuOVGP1, immunoprecipitation experiments revealed similar levels of tyrosine-phosphorylated AKAP3 regardless of the presence or absence of rHuOVGP1. It is noteworthy to mention that previous studies in bovine sperm have shown that tyrosine-phosphorylated AKAP3 was degraded through proteasomal degradation following 4 h of incubation in the capacitation medium [58, 59]. However, in our present study using human sperm, we did not observe significant alterations of the protein level of AKAP3 up to 4 h of capacitation in the presence or absence of rHuOVGP1. Whether rHuOVGP1 exerts a positive influence on enhancement of the level of tyrosine phosphorylation of AKAP3 during capacitation needs to be confirmed in the future using a larger sample size, especially in view of the well-documented fact that large intra- and inter-individual variability exists in various parameters of human sperm [60]. Since enhancement of tyrosine phosphorylation is one of the most important physiological changes during sperm capacitation, it will be of interest in the future to utilize high-throughput phosphoproteome analysis to investigate the effect of rHuOVGP1 on the overall phosphorylation events during sperm capacitation in humans. Phosphoproteome analysis has been used to identify and characterize tyrosine phosphoproteins during capacitation in human sperm, including AKAP3 and AKAP4, and mapped eight phosphorylation sites of these proteins [53]. This approach has been used to study phosphoprotein alterations in sperm between healthy and asthenospermic individuals and between capacitated and non-capacitated sperm [61]. Therefore, phosphoproteomic analysis with high-throughput ability will be a useful tool for determining the effect of rHuOVGP1 on tyrosine phosphorylation of sperm proteins during capacitation.

The FS is a cytoskeletal structure unique to sperm that surrounds the ODF and axoneme in the principal piece of the sperm tail [62]. Functionally, FS provides a scaffold for glycolytic and signaling enzymes during capacitation that is important for hyperactivated motility [62–64]. AKAP3 has been reported to localize only to the principal piece region of mouse sperm [65, 66]. In human sperm, antibodies against variants of AKAP3 have detected disparate localization patterns in head and tail structures [45, 67]. The present study, using an antibody against the C-terminal region of AKAP3, is the first to show the localization of AKAP3 to both acrosomal and tail regions of human sperm, which corroborated results previously found in bovine sperm [46]. Our double immunolabeling experiment of pY proteins and AKAP3 showed that only AKAP3 in the principal piece and the neck region was co-localized with pY proteins while AKAP3 in the acrosomal region and mid-piece was not. One possible explanation for this difference in co-localization of pY proteins and AKAP3 between the principle piece/neck region and the acrosome reaction could be that human AKAP3 retains the dual-binding specificity properties found in the mouse sperm where AKAP3 binds to both PKA regulatory subunits, the RI, which is present in the acrosomal region, and the RII, which is exclusively found in the tail region [68].

The present study also showed the binding of rHuOVGP1 to the ZP of human oocytes, which is consistent with results previously reported by the use of partially purified native human OVGP1 [31]. Importantly, the number of sperm bound to the ZP was significantly increased in the presence of rHuOVGP1. Pre-treatment of either sperm or hemizonae alone with rHuOVGP1 increased the number of sperm bound to hemizonae. However, pre-treatment of both sperm and hemizonae prior to co-incubation yielded the highest increase of the HZI by 2.37-fold. The present results indicate that OVGP1 possesses dual-binding specificity towards sperm and zona pellucida resulting in the enhancement of sperm capacitation as well as sperm-oocyte binding. The presence of rHuOVGP1 in the incubating medium during the co-incubation of sperm and oocytes represents the optimal condition and mimics the in vivo situation where sperm fertilize the oocyte in the oviduct. Our results are consistent with studies conducted in the hamster [35] and porcine [69] where recombinant OVGP1 of the respective species was found to bind to the ZP and enhance sperm-egg binding. In the porcine, the N-terminal region of recombinant OVGP1 is responsible for the binding of OVGP1 to the ZP while the C-terminal region influences the capacity of the full-length OVGP1 in penetrating the ZP and its sensitivity to pronase digestion as well as the subsequent fertilization efficiency [69]. Different functional properties of the N-terminus and C-terminus of rHuOVGP1 remain to be elucidated.

## Conclusion

Results from the present study showed that, during human sperm capacitation, tyrosine-phosphorylated proteins are predominantly located in the FS. AKAP3 has been detected in the acrosome, neck region, mid-piece, and principal piece of the human sperm tail. AKAP3 co-migrates with the tyrosine-phosphorylated p105. Double labeling experiments indicate that AKAP3 in the neck region and principal piece are co-localized with tyrosine-phosphorylated proteins whereas AKAP3 in the acrosomal region and mid-piece is not. Taken together, results from our previous study [36] and the present findings suggest that rHuOVP1 enhances sperm capacitation, in part, through the increase in the level of tyrosine phosphorylation of sperm proteins that are associated with AKAP3 in the sperm tail. In vitro experiments in the present study showed that rHuOVGP1 binds to the ZP of human oocytes and increases sperm-zona binding. Based on these results, supplement of rHuOVGP1 in capacitating medium in standard in vitro fertilization procedures could be beneficial for increasing the fertilization success rate. Failed sperm binding and lack of fertilization is a major limitation of human IVF therapy mainly due to male infertility indications. Inclusion of rHuOVGP1 in the capacitating medium may be particularly useful for treatment of male infertility with mild cases of male factor. What remains to be studied in the future is the mechanism of rHuOVGP1 that regulates the increase in tyrosine phosphorylation of sperm proteins during capacitation and the enhancement of human sperm-egg binding.

## References

[1] Hunter RH (2003). Reflections upon sperm-endosalpingeal and sperm-zona pellucida interactions in vivo and in vitro. Reprod Domest Anim.

[2] Kapur RP, Johnson LV (1985). An oviductal fluid glycoprotein associated with ovulated mouse ova and early embryos. Dev Biol.

[3] Kapur RP, Johnson LV (1986). Selective sequestration of an oviductal fluid glycoprotein in the perivitelline space of mouse oocytes and embryos. J Exp Zool.

[4] Leveille MC, Roberts KD, Chevalier S, Chapdelaine A, Bleau G (1987). Uptake of an oviductal antigen by the hamster zona pellucida. Biol Reprod.

[5] Oliphant G, Ross PR (1982). Demonstration of production and isolation of three sulfated glycoproteins from the rabbit oviduct. Biol Reprod.

[6] Oliphant G, Reynolds AB, Smith PF, Ross PR, Marta JS (1984). Immunocytochemical localization and determination of hormone-induced synthesis of the sulfated oviductal glycoproteins. Biol Reprod.

[7] Saint-Dizier M, Marnier C, Tahir MZ, Grimard B, Thoumire S, Chastant-Maillard S, Reynaud K (2014). OVGP1 is expressed in the canine oviduct at the time and place of oocyte maturation and fertilization. Mol Reprod Dev.

[8] Hachen A, Jewgenow K, Braun BC (2012). Sequence analysis of feline oviductin and its expression during the estrous cycle in the domestic cat (*Felis catus*). Theriogenology..

[9] Sutton R, Nancarrow CD, Wallace AL, Rigby NW (1984). Identification of an oestrus-associated glycoprotein in oviducal fluid of the sheep. J Reprod Fertil.

[10] Sutton R, Nancarrow CD, Wallace AL (1986). Oestrogen and seasonal effects on the production of an oestrus-associated glycoprotein in oviducal fluid of sheep. J Reprod Fertil.

[11] Gandolfi F, Brevini TA, Richardson L, Brown CR, Moor RM (1989). Characterization of proteins secreted by sheep oviduct epithelial cells and their function in embryonic development. Development.

[12] Buhi WC, Alvarez IM, Sudhipong V, Dones-Smith MM (1990). Identification and characterization of de novo-synthesized porcine oviductal secretory proteins. Biol Reprod.

[13] Malayer JR, Hansen PJ, Buhi WC (1988). Secretion of proteins by cultured bovine oviducts collected from estrus through early diestrus. J Exp Zool.

[14] Boice ML, Geisert RD, Blair RM, Verhage HG (1990). Identification and characterization of bovine oviductal glycoproteins synthesized at estrus. Biol Reprod.

[15] Verhage HG, Mavrogianis PA, Boomsma RA, Schmidt A, Brenner RM, Slayden OV, Jaffe RC (1997). Immunologic and molecular characterization of an estrogen-dependent glycoprotein in the rhesus (*Macaca mulatta*) oviduct. Biol Reprod.

[16] Fazleabas AT, Verhage HG (1986). The detection of oviduct-specific proteins in the baboon (*Papio anubis*). Biol Reprod.

[17] Verhage HG, Boice ML, Mavrogianis P, Donnelly K, Fazleabas AT (1989). Immunological characterization and immunocytochemical localization of oviduct-specific glycoproteins in the baboon (*Papio anubis*). Endocrinology..

[18] Verhage HG, Fazleabas AT, Donnelly K (1988). The in vitro synthesis and release of proteins by the human oviduct. Endocrinology..

[19] Aviles M, Gutierrez-Adan A, Coy P (2010). Oviductal secretions: will they be key factors for the future ARTs?. Mol Hum Reprod.

[20] Buhi WC, Bazer FW, Alvarez IM, Mirando MA (1991). In vitro synthesis of oviductal proteins associated with estrus and 17 beta-estradiol-treated ovariectomized ewes. Endocrinology.

[21] Murray MK (1993). An estrogen-dependent glycoprotein is synthesized and released from the oviduct in a temporal- and region-specific manner during early pregnancy in the ewe. Biol Reprod.

[22] Arias EB, Verhage HG, Jaffe RC (1994). Complementary deoxyribonucleic acid cloning and molecular characterization of an estrogen-dependent human oviductal glycoprotein. Biol Reprod.

[23] Malette B, Filion B, St-Jacques S, Kan FW, Bleau G (1995). Hormonal control of the biosynthesis of hamster oviductin. Microsc Res Tech.

[24] Briton-Jones C, Lok IH, Yuen PM, Chiu TT, Cheung LP, Haines C (2001). Regulation of human oviductin mRNA expression in vivo. Fertil Steril.

[25] Briton-Jones C, Lok IH, Cheung CK, Chiu TT, Cheung LP, Haines C (2004). Estradiol regulation of oviductin/oviduct-specific glycoprotein messenger ribonucleic acid expression in human oviduct mucosal cells in vitro. Fertil Steril.

[26] McBride DS, Boisvert C, Bleau G, Kan FW (2004). Evidence for the regulation of glycosylation of golden hamster (*Mesocricetus auratus*) oviductin during the estrous cycle. Biol Reprod.

[27] Chen S, Einspanier R, Schoen J (2013). *In vitro* mimicking of estrous cycle stages in porcine oviduct epithelium cells: estradiol and progesterone regulate differentiation, gene expression, and cellular function. Biol Reprod.

[28] Abe H, Sendai Y, Satoh T, Hoshi H (1995). Bovine oviduct-specific glycoprotein: a potent factor for maintenance of viability and motility of bovine spermatozoa in vitro. Mol Reprod Dev.

[29] King RS, Anderson SH, Killian GJ (1994). Effect of bovine oviductal estrus-associated protein on the ability of sperm to capacitate and fertilize oocytes. J Androl.

[30] Boatman DE, Magnoni GE (1995). Identification of a sperm penetration factor in the oviduct of the golden hamster. Biol Reprod.

[31] O’Day-Bowman MB, Mavrogianis PA, Reuter LM, Johnson DE, Fazleabas AT, Verhage HG (1996). Association of oviduct-specific glycoproteins with human and baboon (Papio anubis) ovarian oocytes and enhancement of human sperm binding to human hemizonae following in vitro incubation. Biol Reprod.

[32] Kouba AJ, Abeydeera LR, Alvarez IM, Day BN, Buhi WC (2000). Effects of the porcine oviduct-specific glycoprotein on fertilization, polyspermy, and embryonic development in vitro. Biol Reprod.

[33] Hill JL, Wade MG, Nancarrow CD, Kelleher DL, Boland MP (1997). Influence of ovine oviducal amino acid concentrations and an ovine oestrus-associated glycoprotein on development and viability of bovine embryos. Mol Reprod Dev.

[34] Saccary L, She YM, Oko R, Kan FW (2013). Hamster oviductin regulates tyrosine phosphorylation of sperm proteins during in vitro capacitation. Biol Reprod.

[35] Yang X, Zhao Y, Yang X, Kan FW (2015). Recombinant hamster oviductin is biologically active and exerts positive effects on sperm functions and sperm-oocyte binding. PLoS One.

[36] Zhao Y, Yang X, Jia Z, Reid RL, Leclerc P, Kan FW (2016). Recombinant human oviductin regulates protein tyrosine phosphorylation and acrosome reaction. Reproduction..

[37] WHO (2010). WHO laboratory manual for the examination and processing of human semen, 5th edn.

[38] Oehninger S, Morshedi M, Franken D (2013). The hemizona assay for assessment of sperm function. Methods Mol Biol.

[39] Luconi M, Porazzi I, Ferruzzi P, Marchiani S, Forti G, Baldi E (2005). Tyrosine phosphorylation of the a kinase anchoring protein 3 (AKAP3) and soluble adenylate cyclase are involved in the increase of human sperm motility by bicarbonate. Biol Reprod.

[40] Visconti PE, Moore GD, Bailey JL, Leclerc P, Connors SA, Pan D, Olds-Clarke P, Kopf GS (1995). Capacitation of mouse spermatozoa. II. Protein tyrosine phosphorylation and capacitation are regulated by a cAMP-dependent pathway. Development..

[41] Bailey JL (2010). Factors regulating sperm capacitation. Syst Biol Reprod Med.

[42] Signorelli J, Diaz ES, Morales P (2012). Kinases, phosphatases and proteases during sperm capacitation. Cell Tissue Res.

[43] Carrera A, Gerton GL, Moss SB (1994). The major fibrous sheath polypeptide of mouse sperm: structural and functional similarities to the A-kinase anchoring proteins. Dev Biol.

[44] Fulcher KD, Mori C, Welch JE, O’Brien DA, Klapper DG, Eddy EM (1995). Characterization of Fsc1 cDNA for a mouse sperm fibrous sheath component. Biol Reprod.

[45] Mandal A, Naaby-Hansen S, Wolkowicz MJ, Klotz K, Shetty J, Retief JD, Coonrod SA, Kinter M, Sherman N, Cesar F, Flickinger CJ, Herr JC (1999). FSP95, a testis-specific 95-kilodalton fibrous sheath antigen that undergoes tyrosine phosphorylation in capacitated human spermatozoa. Biol Reprod.

[46] Vijayaraghavan S, Liberty GA, Mohan J, Winfrey VP, Olson GE, Carr DW (1999). Isolation and molecular characterization of AKAP110, a novel, sperm-specific protein kinase A-anchoring protein. Mol Endocrinol.

[47] Baker MA, Reeves G, Hetherington L, Aitken RJ (2010). Analysis of proteomic changes associated with sperm capacitation through the combined use of IPG-strip pre-fractionation followed by RP chromatography LC-MS/MS analysis. Proteomics.

[48] Sakkas D, Leppens-Luisier G, Lucas H, Chardonnens D, Campana A, Franken DR, Urner F (2003). Localization of tyrosine phosphorylated proteins in human sperm and relation to capacitation and zona pellucida binding. Biol Reprod.

[49] Oko R (1988). Comparative analysis of proteins from the fibrous sheath and outer dense fibers of rat spermatozoa. Biol Reprod.

[50] Petersen C, Füzesi L, Hoyer-Fender S (1999). Outer dense fibre proteins from human sperm tail: molecular cloning and expression analyses of two cDNA transcripts encoding proteins of approximately 70 kDa. Mol Hum Reprod.

[51] Baker MA, Smith ND, Hetherington L, Taubman K, Graham ME, Robinson PJ, Aitken RJ (2010). Label-free quantitation of phosphopeptide changes during rat sperm capacitation. J Proteome Res.

[52] Mariappa D, Aladakatti RH, Dasari SK, Sreekumar A, Wolkowicz M, Van Der HF, Seshagiri PB (2010). Inhibition of tyrosine phosphorylation of sperm flagellar proteins, outer dense fiber protein-2 and tektin-2, is associated with impaired motility during capacitation of hamster spermatozoa. Mol Reprod Dev.

[53] Ficarro S, Chertihin O, Westbrook VA, White F, Jayes F, Kalab P, Marto JA, Shabanowitz J, Herr JC, Hunt DF, Visconti PE (2003). Phosphoproteome analysis of capacitated human sperm. Evidence of tyrosine phosphorylation of a kinase-anchoring protein 3 and valosin-containing protein/p97 during capacitation. J Biol Chem.

[54] Nixon B, Bielanowicz A, Anderson AL, Walsh A, Hall T, McCloghry A, Aitken RJ (2010). Elucidation of the signaling pathways that underpin capacitation-associated surface phosphotyrosine expression in mouse spermatozoa. J Cell Physiol.

[55] Miki K, Eddy EM (1998). Identification of tethering domains for protein kinase A type Ialpha regulatory subunits on sperm fibrous sheath protein FSC1. J Biol Chem.

[56] Michel JJ, Scott JD (2002). AKAP mediated signal transduction. Annu Rev Pharmacol Toxicol.

[57] Tasken K, Aandahl EM (2004). Localized effects of cAMP mediated by distinct routes of protein kinase A. Physiol Rev.

[58] Hillman P, Ickowicz D, Vizel R, Breitbart H (2013). Dissociation between AKAP3 and PKARII promotes AKAP3 degradation in sperm capacitation. PLoS One.

[59] Vizel R, Hillman P, Ickowicz D, Breitbart H, Biochim Biophys A (2015). AKAP3 degradation in sperm capacitation is regulated by its tyrosine phosphorylation. Biochim Biophys Acta.

[60] Overstreet JW (1994). Clinical approach to male reproductive problems. Occup Med.

[61] Cao X, Cui Y, Zhang X, Lou J, Zhou J, Bei H, Wei R (2018). Proteomic profile of human spermatozoa in healthy and asthenozoospermic individuals. Reprod Biol Endocrinol.

[62] Eddy EM, Toshimori K, O’Brien DA (2003). Fibrous sheath of mammalian spermatozoa. Microsc Res Tech.

[63] Miki K, Willis WD, Brown PR, Goulding EH, Fulcher KD, Eddy EM (2002). Targeted disruption of the Akap4 gene causes defects in sperm flagellum and motility. Dev Biol.

[64] Krisfalusi M, Miki K, Magyar PL, O’Brien DA (2006). Multiple glycolytic enzymes are tightly bound to the fibrous sheath of mouse spermatozoa. Biol Reprod.

[65] Brown RL, August SL, Williams CJ, Moss SB (2003). AKAP7gamma is a nuclear RI-binding AKAP. Biochem Biophys Res Commun.

[66] Xu Y, Xie J, Chen R, Cao Y, Ping Y, Xu Q, Hu W, Wu D, Gu L, Zhou H, Chen X, Zhao Z, Zhong J, Li R (2016). Fluorescence- and magnetic-activated cell sorting strategies to separate spermatozoa involving plural contributors from biological mixtures for human identification. Sci Rep.

[67] Lefevre A, Duquenne C, Rousseau-Merck MF, Rogier E, Finaz C (1999). Cloning and characterization of SOB1, a new testis-specific cDNA encoding a human sperm protein probably involved in oocyte recognition. Biochem Biophys Res Commun.

[68] Xu K, Qi H (2014). Sperm-specific AKAP3 is a dual-specificity anchoring protein that interacts with both protein kinase a regulatory subunits via conserved N-terminal amphipathic peptides. Mol Reprod Dev.

[69] Algarra B, Han L, Soriano-Ubeda C, Aviles M, Coy P, Jovine L, Jimenez-Movilla M (2016). The C-terminal region of OVGP1 remodels the zona pellucida and modifies fertility parameters. Sci Rep.

